# A personalized tumor kV‐IORT navigation system based on hybrid twin

**DOI:** 10.1002/acm2.70243

**Published:** 2025-09-19

**Authors:** Shan Jiang, Shuo Yang, Zhiyong Yang, Daguang Zhang, Yingkai Luan, Zeyang Zhou

**Affiliations:** ^1^ School of Mechanical Engineering Tianjin University Tianjin China; ^2^ Department of Radiation Oncology Tianjin Medical University Cancer Institute & Hospital Tianjin China

**Keywords:** holographic dose, hybrid twin, kV‐IORT, radiotherapy navigation

## Abstract

**Purpose:**

This study proposes a multidimensional hybrid twin navigation system to enhance applicator placement accuracy in low‐energy x‐ray intraoperative radiotherapy (kV‐IORT), particularly for less‐experienced practitioners. The system integrates augmented reality (AR) and digital twin technologies to improve intraoperative precision and usability.

**Methods:**

The system comprises three core modules: (1) a holographic dose (Holo‐dose) model visualizing clinically relevant radiation layers based on Zeiss TARGIT data, (2) a patient‐specific applicator positioning strategy with real‐time AR feedback adapted to tumor bed geometry, and (3) a bidirectional hybrid twin control linking a physical radiotherapy robot with its virtual twin for precise navigation. Phantom experiments utilized a 20 mm spherical applicator within a 30 mm tumor bed boundary. Target dose range was 18.50–19.50 Gy (15 mm radius) over 25 min. Accuracy was evaluated via root mean square error (RMSE) and target registration error (TRE). Mean RMSE was 0.383 mm (phantom) and 0.106 mm (robot); mean TRE was 0.41 mm.

**Results:**

The system significantly enhanced positioning accuracy for novice users. Medical students achieved an average precision of 2.971 mm (*p* = 0.00015), and inexperienced physicians reached 2.803 mm (*p* = 0.00038). No significant improvement was observed for experienced surgeons (*p* > 0.05), indicating the system's potential as a training and assistance tool. The average System Usability Scale (SUS) score was 83.5, suggesting high user satisfaction, especially among younger participants.

**Conclusions:**

The hybrid twin navigation system significantly improved applicator placement accuracy for novice users, demonstrating its value as an effective training and assistance tool for kV‐IORT. High user satisfaction and sub‐millimeter registration and alignment accuracy confirm its potential for clinical translation, particularly in enhancing usability and precision for less‐experienced practitioners.

## 1 INTRODUCTION

X‐ray intraoperative radiotherapy (kV‐IORT) using low‐energy x‐rays has become an effective method for the elimination of hidden microscopic tumor cells around the surgical cavity following tumor resection.^1^ kV‐IORT can deliver a single large dose of radiation, precisely targeting areas with the highest risk of recurrence while minimizing damage to surrounding healthy tissues.^2^

INTRABEAM (Carl Zeiss Meditec, Inc., Oberkochen, Germany) is a dedicated electronic brachytherapy system widely used in the field of kV‐IORT.^3^ This system features a terminal x‐rays probe capable of emitting photon beams in the range of 30–50 kV. However, clinical practice indicates that only 50 kV x‐rays are used to irradiate the tumor bed during kV‐IORT.^4^, ^5^ During irradiation, the radiation applicator is typically fixed at the end of the probe, with different types of applicators used for tumor beds located in various anatomical regions of the patient's body. Spherical applicators are often employed for certain hollow spherical surgical cavities, such as those found in breast cancer and brain metastases.^6^, ^7^ The diameter of spherical applicator ranges from 1.5 to 5 cm,^8^ and during kV‐IORT, the appropriate size applicator is chosen based on the volume of the surgical cavity, ensuring complete coverage of the tumor bed's surface.

Recent seminal work by Lozares et al.^9^ has established critical protocols for surface dose optimization in low‐energy IORT through rigorous in vivo dosimetry studies, demonstrating rapid dose attenuation gradients that directly inform spatial control strategies. However, the process of placing the spherical applicator into the surgical cavity is somewhat random. It typically relies on a preoperative empirical assessment of the tumor bed's location based on processed computed tomography (CT) images, which may not accurately determine the relative positioning of the applicator to the tumor bed while considering the duration of kV‐IORT. This can lead to inadequate radiation dosage delivery, increasing the risk of postoperative recurrence.^10^ Therefore, precise intraoperative localization of the applicator and stringent control of the radiation delivery time are critical.

In recent years, augmented reality (AR) and mixed reality (MR) have been widely applied to assist intraoperative navigation, aiming to enhance surgical accuracy and safety.^11^ For example, Ambrosini et al.^12^ demonstrated a system that tracks probes in 3D space to assist surgeons in better locating tumors during resection surgeries. Trojak et al.^13^ developed a mixed reality navigation system for percutaneous biopsy that allows for precise needle placement without physical markers, reducing the number of punctures required to reach the target and shortening surgery time. Zhou et al.^14^ created a mixed reality navigation system for facilitating brachytherapy, successfully integrating medical imaging with preoperative planning to help clinicians locate needles more efficiently. Building on these innovations, Lozares‐Cordero et al.^15^ validated a photogrammetry‐based IORT navigation framework through 3D‐printed tumor bed reconstructions and radiochromic film dosimetry—a pivotal advancement enabling intraoperative surface reconstruction and spatial parameter integration. Perkins et al.^16^ proposed using mixed reality technology to accurately register preoperative magnetic resonance imaging (MRI) scans to the patient's breast, assisting surgeons in tumor localization during breast cancer resection procedures.

In summary, most intraoperative navigation studies enhance target localization accuracy by mapping medical images to patients, with limited focus on digital interaction based on the specificity of surgical instruments and target areas. Notably, there has been little specific research on three‐dimensional imaging‐guided kV‐IORT navigation. Complementary studies by Lozares‐Cordero et al.^17^ in gynecologic brachytherapy further substantiate electronic sources' dosimetric efficacy, providing critical validation of kV‐IORT's capacity to balance therapeutic goals with organ‐at‐risk protection. To enable real‐time digital interaction between the terminal applicator and the tumor bed for precise radiation dosage delivery in kV‐IORT, the concept of hybrid twin has been introduced. Hybrid twin, as a level of AR‐assisted digital twins, emphasizes virtual‐to‐entity analysis and feedback, including visual registration, multimodal interaction and control, and the functionalities derived from these processes.^18^, ^19^

Therefore, we propose a multidimensional hybrid twin navigation system for kV‐IORT, which offers three key innovations: deep coupling of dose distribution models with AR spatial rendering; a multidimensional interaction paradigm integrating robotic, artifact, and physician cognition; and a bidirectional driving mechanism based on joint angle feedback. These innovations enhance spatial accuracy, real‐time dose‐response, and human‐machine collaboration. The system enables precise control of the robot's applicator, ensuring optimal positioning above the tumor bed for accurate dose delivery. Surgeons can use real‐time feedback to precisely determine applicator placement, ensuring effective radiation therapy. Our contributions are divided into two main parts:

1. Personalized adaptive applicator positioning strategy for tumor bed areas. We propose a digital model and spatial mapping system for the kV‐IORT spherical applicator, tailored to the tumor bed. By integrating real‐time feedback from the tumor bed, this method allows precise, efficient positioning of the radiation therapy robot's terminal applicator.
2. Multidimensional integrated hybrid twin navigation mode for kV‐IORT. This navigation system combines surgeon cognition, robot posture, and holographic dosage effects to provide progressive, multidimensional guidance. A hybrid twin model integrates visual and positional feedback, facilitating real‐time correction of the applicator's position. The bidirectional driving model enhances spatial accuracy and optimizes applicator placement, ensuring effective kV‐IORT application.

## 2 HOLO‐DOSE MODEL CONSTRUCTION AND APPLICATOR POSITIONING STRATEGY

### 2.1 Basis for dose construction

Zeiss provides a dose rate table for INTRABEAM users, which presents the percentage depth dose (PDD) for targeted intraoperative radiotherapy (TARGIT) and version 4.0 intraoperative radiotherapy (V4.0), reflecting specific values of dose rate variations with radial distribution from the surface of the spherical applicators. Both dose rates were measured by Zeiss in water phantom experiments and directly input into the INTRABEAM system.^8^ However, research indicates significant differences between the TARGIT and V4.0 dose rates, with the use of V4.0 dose rates posing a risk of inadequate dosing. Therefore, V4.0 is seldom used as a clinical reference for kV‐IORT, with a preference for TARGIT dose rates.^20^, ^21^ Thus, the dose models developed below will reference the standard TARGIT dose rate provided by Zeiss.

**FIGURE 1:**
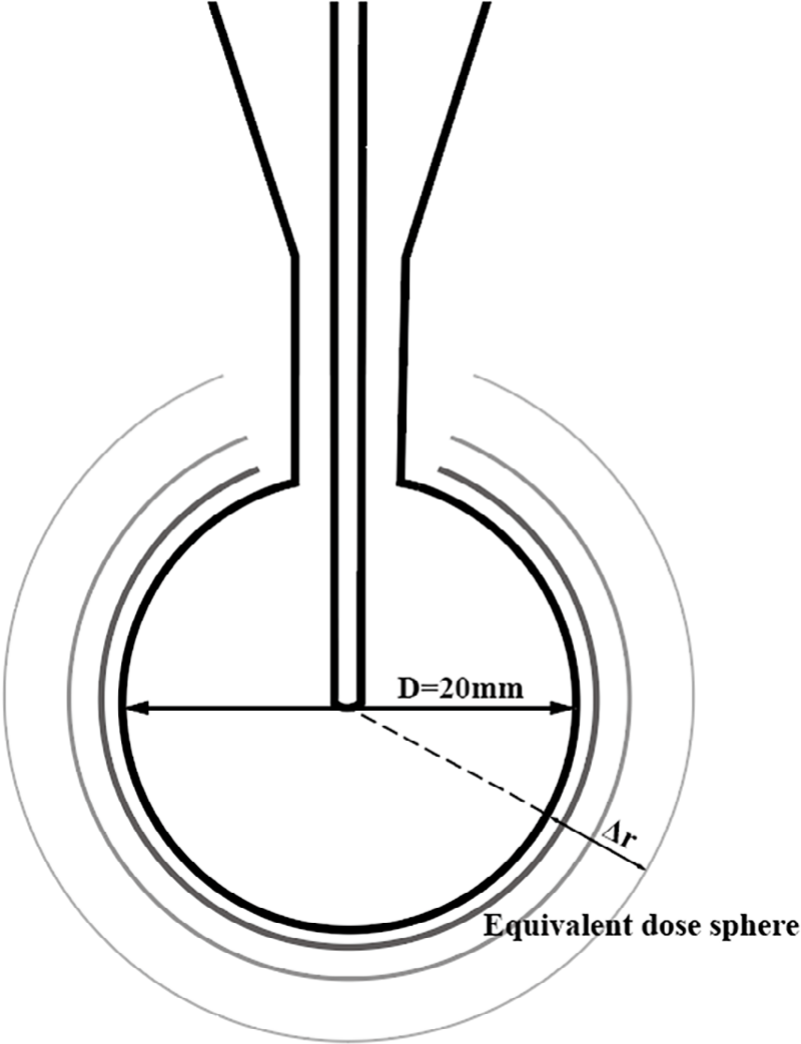
Spherical applicator with a diameter of 20 mm and its equivalent dose sphere.

As mentioned in the introduction, for personalized tumor beds that resemble hollow spheres after partial tissue resection, different types of standard spherical applicators in the INTRABEAM system should be selected. The radiation emitted from the spherical applicator is isotropically divergent and can be viewed as composed of numerous equivalent dose spheres centered at the same point.^22^, ^23^ The further any point on the sphere is from the surface of the applicator (represented by the radial distance Δ*r*, the lower the dose rate becomes. All points on a given equivalent dose sphere have the same dose rate. For instance, with a spherical applicator of 20 mm in diameter, the spherical applicator and its equivalent dose sphere are shown in Figure 1. Building on this, we further determine the effective dose range for irradiating the personalized tumor bed, focusing on two aspects: the delivered dose at the tumor bed surface (*D*_deliver_) and the treatment dose at a depth of 1 cm (*D*_treatment_) from the tumor bed. According to studies on kV‐IORT, and for safety considerations, *D*_deliver_ typically ranges from 18 to‐20 Gy, while *D*_treatment_ ranges from 5 to 7 Gy, with intraoperative irradiation time (*t*) set at 20–35 min.^4^, ^21^

Calculations are conducted under the premise that *D*_deliver_, *D*_treatment_, and *t* remain within the specified safe dose ranges. To clarify the logic behind the dose calculations, we establish nine continuous integer reference irradiation times for each type of spherical applicator, all not less than 20 min.^22^ The Zeiss PDD table provides values for how the dose rate varies with Δ*r*, and the dose is calculated using Equation (1).

(1) $$d_{s}*t_{r}=D_{s}$$

*d*_s_ represents the standard dose rate at all points on a given equivalent dose sphere, *t*_r_ is the reference irradiation time, and *D*_s_ is the calculated standard dose at all points on that equivalent dose sphere for a specific reference time. The initial value of *t*_r_ is set to 20 min. By combining the appropriate *d*_s_ from the Zeiss PDD table, we can calculate the ranges for *D*_deliver_ and *D*_treatment_ when *t*_r_ = 20 min. Similar calculations can be performed for other reference irradiation times *t*_r_, to determine their corresponding dose ranges. The corresponding Zeiss PDD data for the 20 mm spherical applicator are presented in Table 1.

**TABLE 1:** Part of a ZEISS PDD with a 20 mm diameter spherical applicator.

Radial distance(mm)	Dose rate(Gy/min)
0	1.74
0.25	1.65
0.5	1.57
0.75	1.50
1	1.43
1.25	1.37
1.5	1.31
1.75	1.25
2	1.19
2.25	1.14
2.5	1.09

**FIGURE 2:**
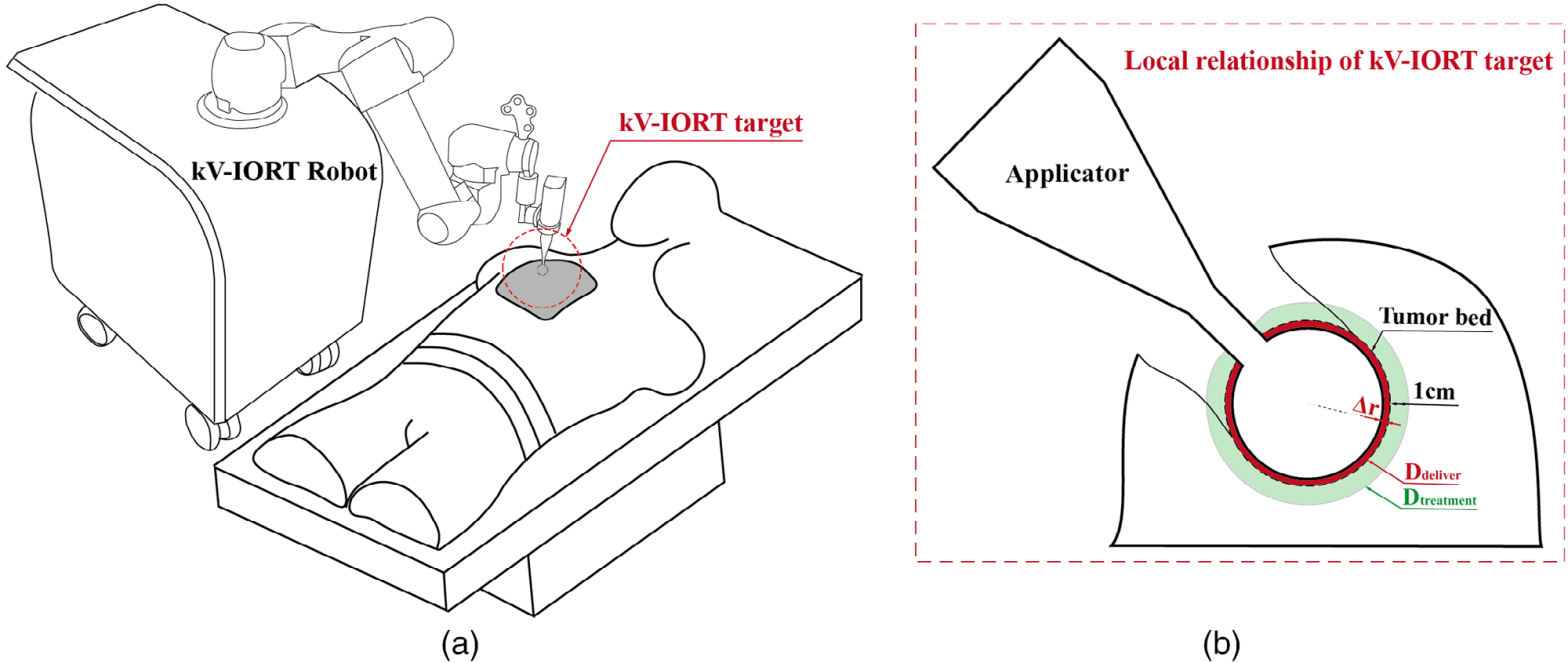
(a) The global relationship between the robot and the patient during kV‐IORT. (b) Spatial position of the applicator relative to the personalized tumor bed in kV‐IORT target (the red border represents the delivered dose on the surface of the tumor bed, the green border represents the treatment dose at a depth of 1 cm from the tumor bed).

If certain models of spherical applicators do not meet the safety dose range when *t*_r_ = 20 min and Δ*r* = 0, the initial value of tr should be appropriately increased according to the safety dose standards. Ultimately, we select the extreme values of *d*_s_ involved in the calculations that meet the specified range for *D*_s_, and reverse‐filter the matching range for Δ*r* from the Zeiss PDD table. The value of Δ*r* constrains the distance between the applicator and the tumor bed surface, which can limit the positioning of the applicator during kV‐IORT. To better understand the irradiation process and calculation logic, Figure 2a,b shows the spatial relationship between the applicator and the personalized tumor bed during kV‐IORT. Detailing the specific parameters involved in the calculations.

Based on this theoretical foundation, Appendix I presents the radial distances and effective dose ranges for various models of spherical applicators (Types with a diameter of 20–40 mm) based on different reference irradiation times.

### 2.2 Method for constructing Holo‐dose model

Vidal et al.^24^ developed a dose calculation tool based on Monte Carlo phase space information, which generates parameterized 2D phase space files suitable for different spherical applicators, accurately predicting and reconstructing dose distributions at various depths in complex tissues. This phase space modeling and prediction method allows for efficient comparison and analysis based on the patient's CT images. However, considering the need for intraoperative applicator positioning relative to the actual tumor bed, providing surgeons with a more straightforward and intuitive 3D dose reference is crucial. Thus, the holographic critical dose layer becomes particularly important.

As mentioned in Section 2.1, the basis for dose construction involves using Unity3D to model the maximum and minimum values within the range of Δ*r* corresponding to a reference time. SolidWorks is utilized to reconstruct simulation models for various spherical applicators. After converting all model files into FBX format using 3ds Max, these files are imported into the designated project in Unity3D. Within the software, model prefabs are created in the hierarchy panel, and simulation material rendering is applied to the models. To reflect the relationship between the critical dose layer and the surface of the spherical applicator, transparent colored sphere objects are added under the prefab to represent the surface dose rates of the critical dose layer. The radius of critical dose sphere is calculated using Equation (2).

(2) $$r_{0}+\Delta r_{x}=R_{x}$$

**FIGURE 3:**
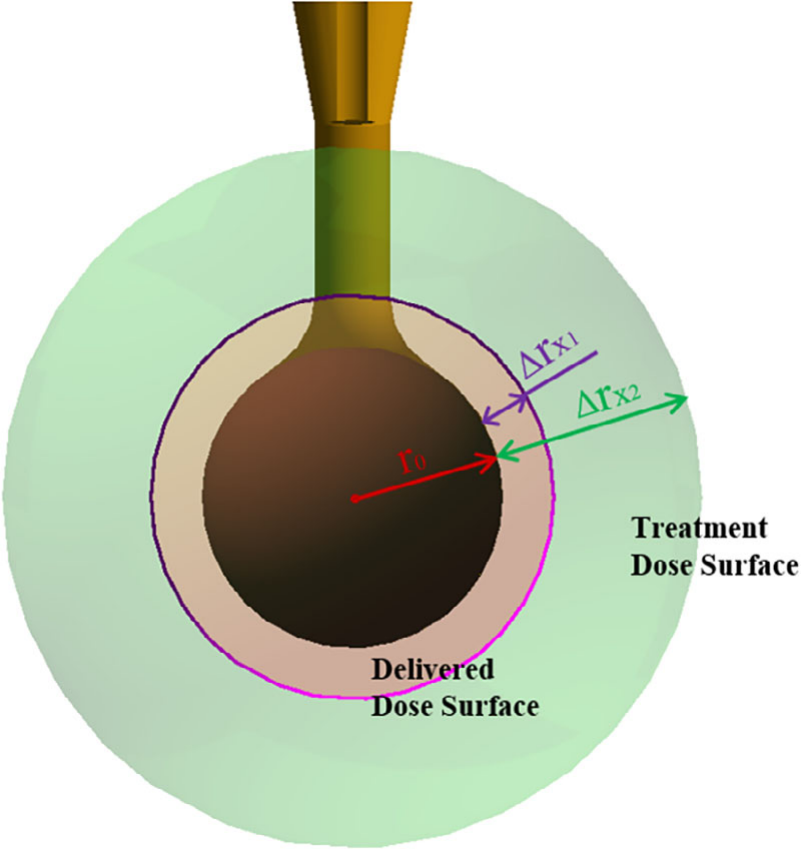
Holo‐dose of the critical dose layer. Holo‐dose, holographic model.

*r*_0_ is the initial diameter of the spherical applicator surface, Δ*r*_x_ is the previously filtered radial length matching value, *R*_x_ is the radius of the critical dose layer sphere object. By establishing the proportional relationship between *R*_x_ and *r*_0_, the dimensions of the critical dose sphere can be determined, allowing for the drawing of a critical dose sphere in Unity3D that shares the same center as the spherical applicator. The constructed holographic model (Holo‐dose) of the critical dose layer is shown in Figure 3. Further deployment of this project in AR device enhances the visualization of the critical dose layer of the spherical applicator.

### 2.3 Applicator positioning method based on personalized tumor bed area adaptation

**FIGURE 4:**
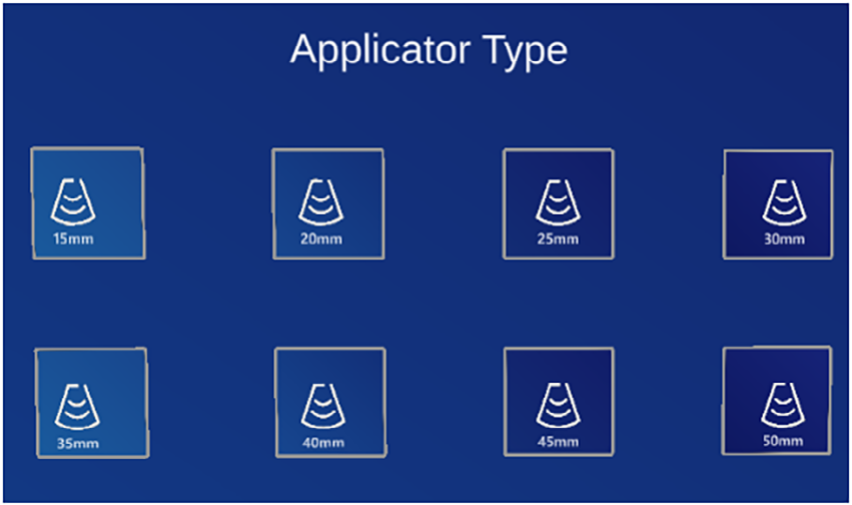
UI hierarchy panel—Applicator's type selection. UI, user interface.

Since surgeons must assess the personalized tumor bed based on the patient's CT images to select the appropriate type of spherical applicator, we constructed an interactive user interface (UI) using the Mixed Reality Toolkit plugin in Unity3D. The UI hierarchy panel for selecting types of the spherical applicators is shown in Figure 4, which includes eight different types for surgeon.

**FIGURE 5:**
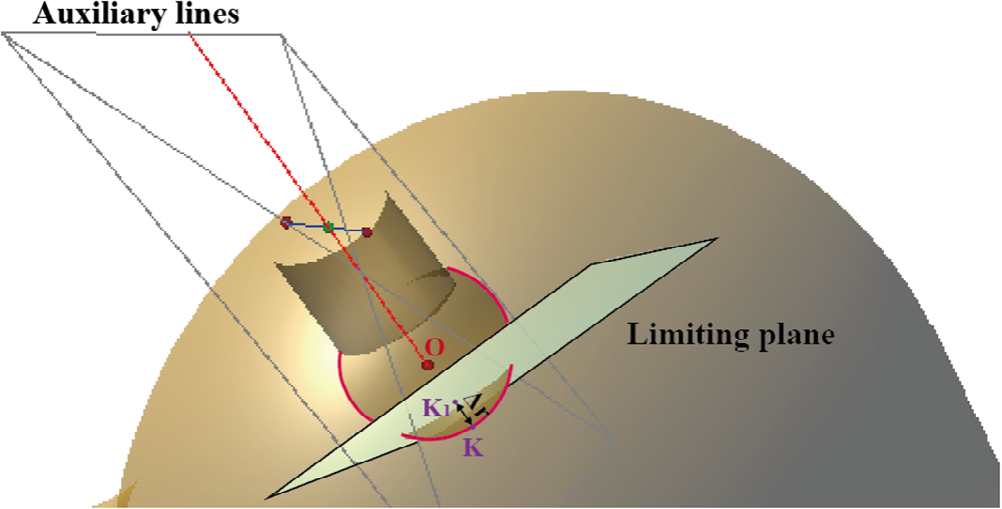
The positional relationship between the spatial limiting plane and the tumor bed.

Based on previous assessment of the personalized tumor bed dimensions, surgeon selects a suitable applicator model from the panel that is smaller than the tumor bed size. The Δ*r* is determined by subtracting the radius of the spherical applicator from that of the tumor bed. A point K is chosen on the tumor bed surface, furthest from the skin incision, and connected to the center O of the spherical applicator. Starting from point K, a distance of Δ*r* is marked along the line segment, and a spatial limiting plane is established at the endpoint K_1_. The final position of the spatial limiting plane relative to the tumor bed is shown in Figure 5.

**FIGURE 6:**
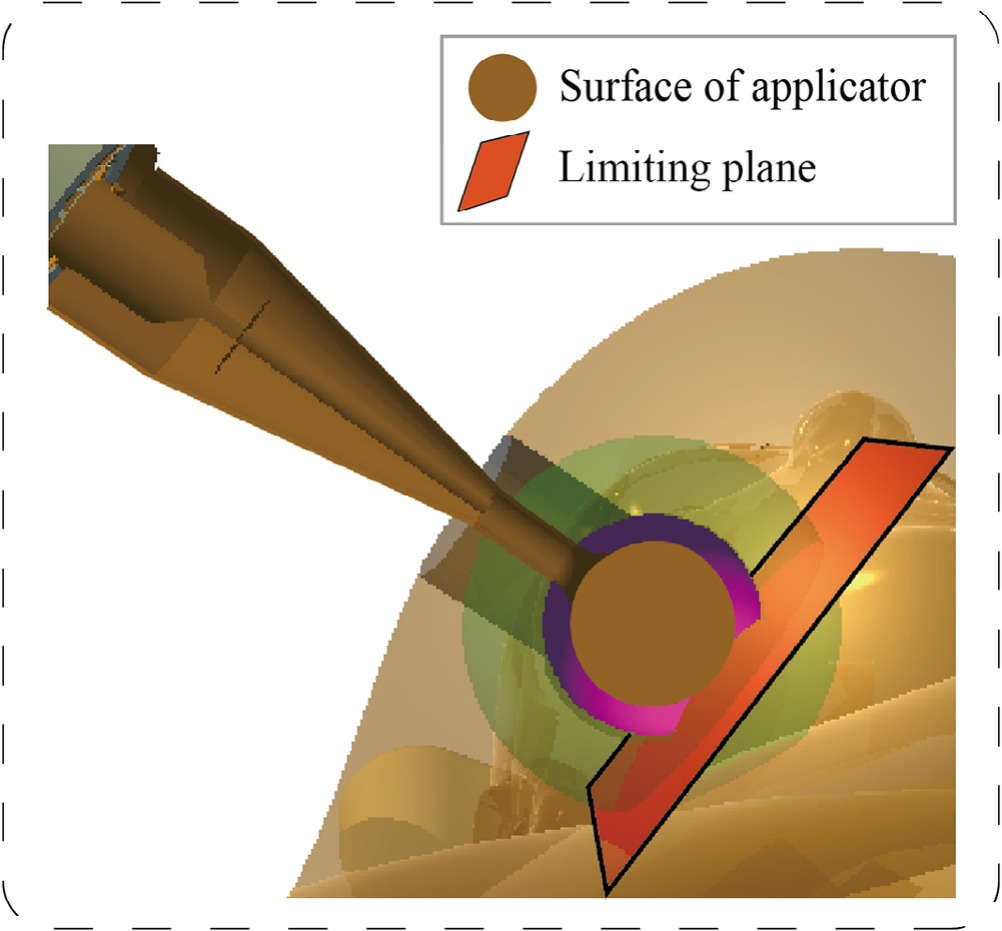
Applicator adapts to the spatial area of tumor bed to achieve positioning effect.

Surgeon moves the model of the spherical applicator in space, positioning the center of the applicator at the geometric center of the reconstructed personalized virtual tumor bed, which has been directly calculated and highlighted in Unity3D. If the applicator is moved to the virtual tumor bed's geometric center, the spatial limiting plane communicates with the surgeon's view by changing color, indicating that the applicator has been placed in the designated position. How the applicator adapts to the spatial area of the personalized tumor bed to achieve effective positioning is shown in Figure 6.

### 2.4 Multidimensional integrated hybrid twin navigation mode for kV‐IORT

#### 2.4.1 A novel navigation system's framework

**FIGURE 7:**
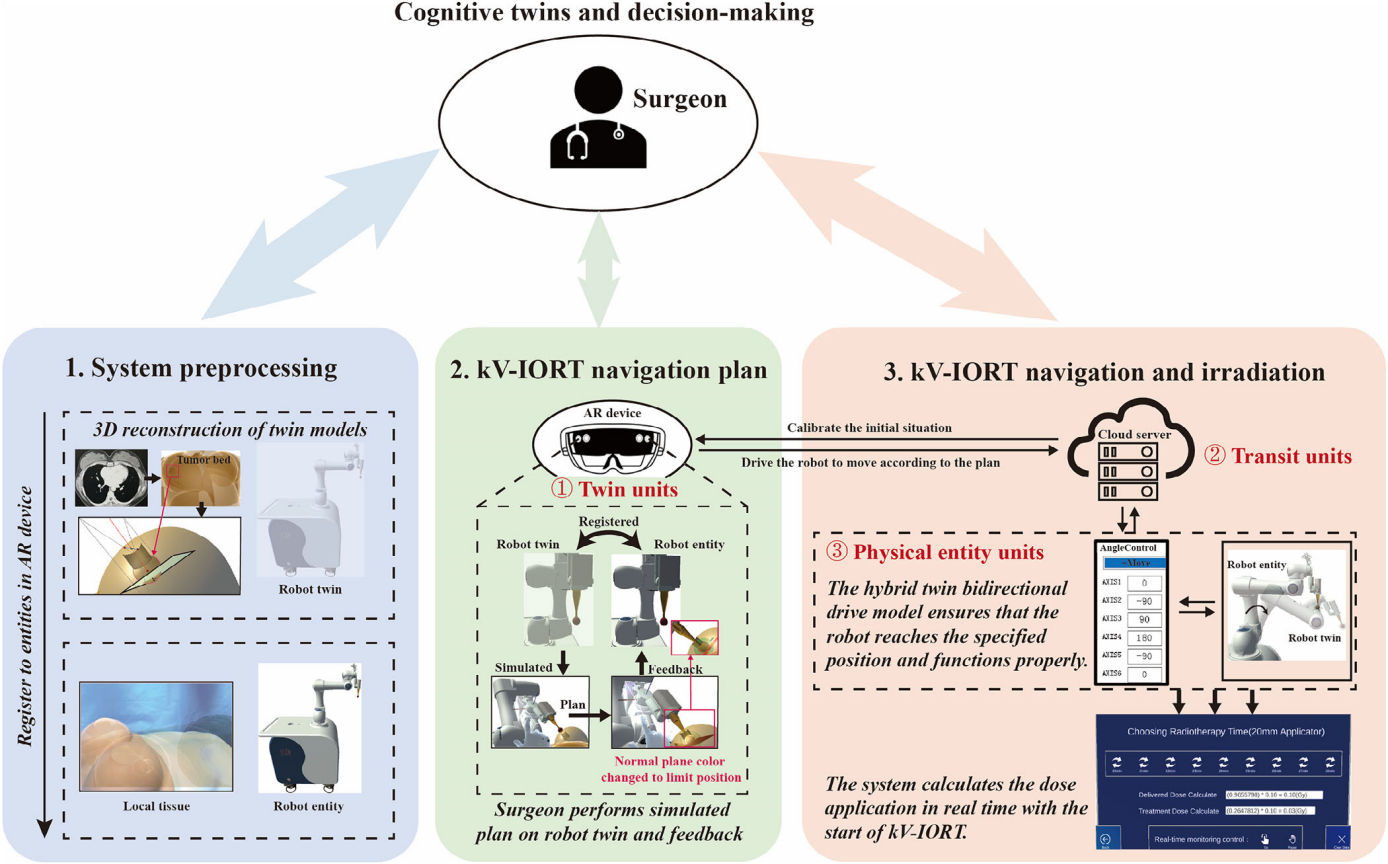
Flowchart of multidimensional fusion hybrid twin radiotherapy navigation.

As shown in Figure 7, we propose a novel navigation system for kV‐IORT, comprising three stages.

In the first stage, system preprocessing is conducted. A 3D reconstruction of the patient's local tissue and the robotic system—collectively referred to as twin models—is generated. Using Mimics (a widely used medical imaging software), the patient's CT images are segmented to extract the overall contour and target area, forming a digital twin of the local tissue. A spherical applicator, pre‐selected based on tumor bed dimensions, is attached to the terminal of the radiation therapy robot. The local tissue twin is imported into Unity3D, where scripts compute the tumor bed's geometric center and limiting planes. The software incorporates complete twin models, registration mechanisms, and digital control modules. Once deployed on AR devices, registration between the digital twins and physical entities can be achieved.

In the second stage, following registration, the surgeon performs kV‐IORT navigation planning. The AR device presents an immersive holographic interface integrating both virtual and physical entities. Guided by clinical cognition, the surgeon interacts with the UI to select the corresponding applicator twin, which connects to the robot twin. The robot twin supports preoperative simulation by allowing adjustment of the end‐effector's position based on cognitive input, establishing a navigation path, and adaptively determining target points using the holographic dose and tumor bed area, thus enabling multidimensional path planning.

In the third stage, real‐time kV‐IORT navigation is executed. The robot's digital control system communicates along the planned path from stage two, guiding the end‐effector of the physical robot to the target site based on the hybrid twin model. If patient positioning changes mid‐procedure, the surgeon must repeat the first two stages to re‐register and update the navigation path. Once the end‐effector reaches the intended position, intraoperative irradiation begins, with real‐time dose monitoring through the interactive UI.

Throughout the navigation process, the multidimensional hybrid twin remains integrated. Based on the surgeon's cognitive input, the system utilizes Holo‐dose data and the spatial characteristics of the tumor bed to provide visual feedback, aiding in end‐effector posture control, rapid path planning, and precise positioning prior to dose delivery. Once in position, the system performs real‐time dose calculations during kV‐IORT, offering accurate timing and dosage parameters. This empowers the surgeon to manage treatment time and dosage with greater precision.

#### 2.4.2 A radiotherapy robot controlling method based on bidirectional hybrid twin‐driven model

**FIGURE 8:**
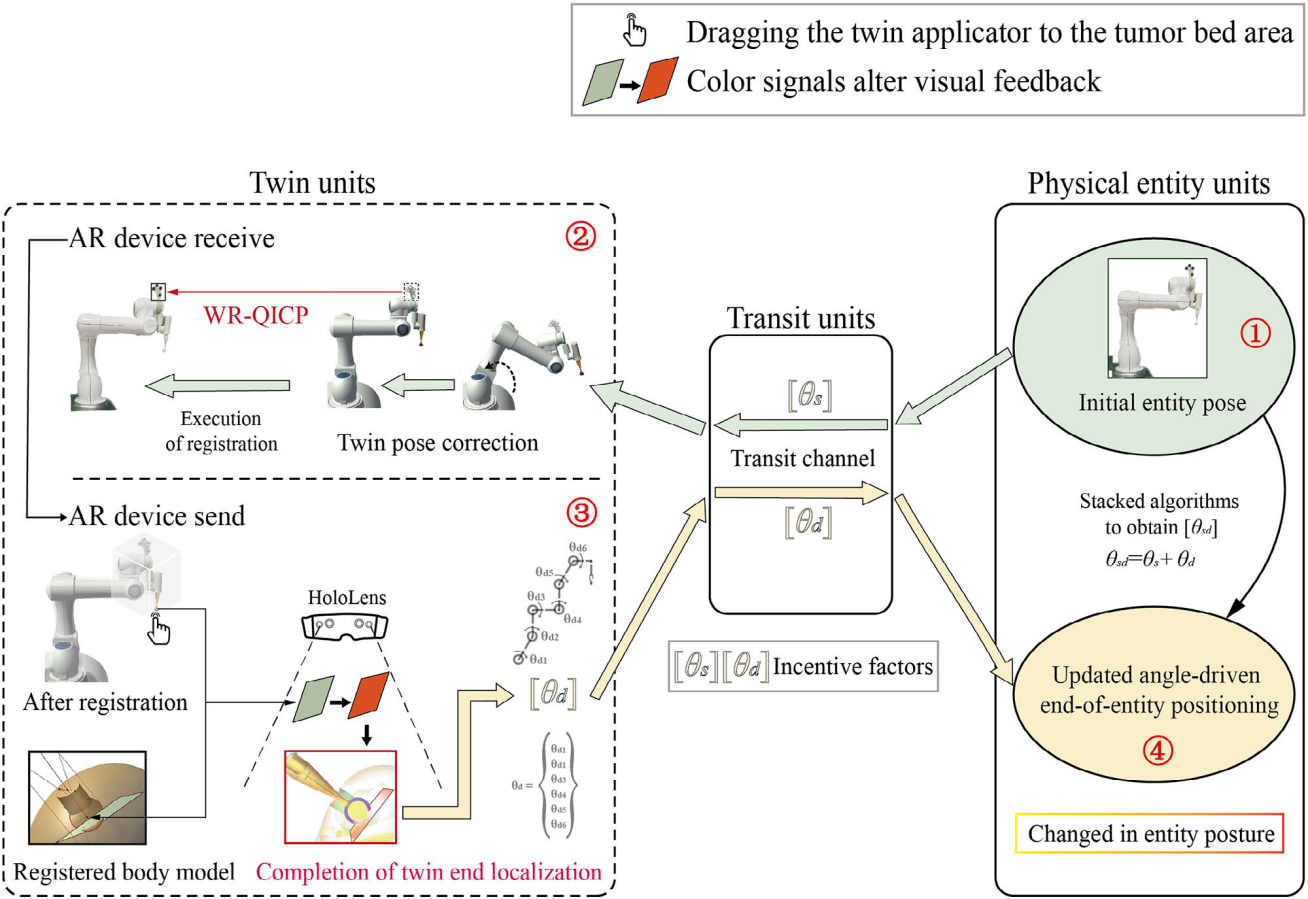
Bidirectional hybrid twin‐driven model framework.

For kV‐IORT navigation, a radiotherapy robot control method based on bidirectional hybrid twin‐driven model was designed to ensure the precise positioning of the radiotherapy robot's end‐effector at the target location. The framework of bidirectional hybrid twin‐driven model is shown in Figure 8.

The bidirectional hybrid twin‐driven model consists of three main components: the twin units, the transit units, and the physical entity units. The twin units include the radiotherapy robot's twin model within the AR holographic environment. The transit units consist of a cloud server and its internal bidirectional communication channel. The physical entity units comprise the physical radiotherapy robot and its motion control program. A digital communication protocol is employed, allowing the physical entity units to asynchronously send or receive signals to and from the twin units. The twin units are equipped with a signal transceiving mechanism on par with the physical entity to ensure signal synchronization. The transit unit effectively connects the twin units and the physical entity units, where the joint angle difference data of the radiotherapy robot serves as a critical driving factor throughout the model. Surgeons can flexibly utilize the bidirectional hybrid twin‐driven model during surgery, enabling the radiotherapy robot to perform end‐effector posture correction and registration from the physical entity to the twin. The model also facilitates the bidirectional driven process from the twin to the physical entity, accurately controlling the end‐effector's applicator placement at the tumor bed.

**FIGURE 9:**
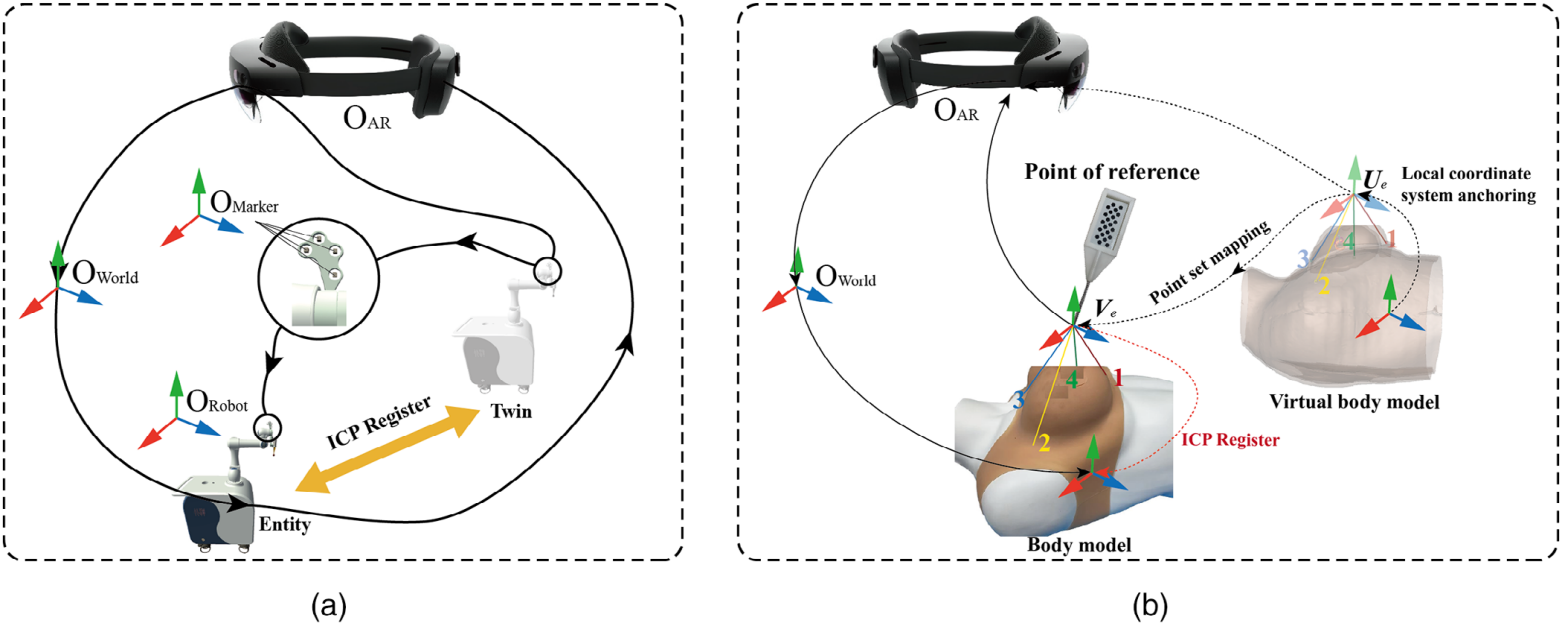
(a) The coordinate relationship between kV‐IORT robot twin and entity registration (the AR device recognizes the four‐point marker on the entity and obtains O_robot_ in the world coordinate system, and the ICP algorithm can find the transformation matrix from the twin four‐point marker O_Marker_ to the entity to complete registration). (b) The coordinate relationship between virtual patient twin and entity registration(The AR device achieved real‐time detection of the probe and acquired the tip positions in both the physical patient coordinate system $V_{e}$ and the virtual twin coordinate system $U_{e}$. The ICP algorithm subsequently calculated their transformation matrix, thereby completing the system registration). AR, augmented reality; ICP, iterative closest point; kV‐IORT, x‐ray intraoperative radiotherapy.

Before registration of the radiotherapy robot, the posture of the twin in AR device is random and often inconsistent with the physical entity. Calibrating the twin to maintain the same posture as the physical entity is a prerequisite for achieving precise registration. In Figure 7, part ③ of the physical entity units’ motion control interface displays the robot's initial six joint angles, *θ*_s._ Following the green arrows in Figure 8, the robot's joint angles *θ*_s_ are converted into string data, transmitted through the physical entity units—transit units—twin units, and applied to each joint of the radiotherapy robot's twin to update its initial posture. In Figure 8, the twin units’ AR device reception section shows the change in posture of the radiotherapy robot twin before and after receiving the initial joint angle information. Once the posture is updated, the iterative closest point (ICP) algorithm‐based on point‐to‐point registration can be used to complete the registration. This step involves mapping the surgeon's arm position into AR environment. As shown in Figure 9, the multi‐module registration method oriented to mixed reality constitutes the critical foundation for virtual‐physical integration. Two registration modules were implemented: (a) demonstrates the registration between the radiotherapy robot twin and its physical counterpart, while (b) illustrates the spatial alignment of reconstructed virtual tumor bed with adjacent skin tissues to the actual patient anatomy. Through these separate registration processes, surgeons could dynamically monitor the spatial relationship between the robot and patient anatomy via synchronized twins, achieving virtual‐physical superposition that enhanced the intuitiveness and predictability of the comprehensive kV‐IORT system.

Additionally, to assist in radiotherapy robot path planning, the Mixed Reality Toolkit is used to construct a draggable bounding box B_1_ on the base coordinate system of the robot's twin, as shown in Figure 8. By using the spatial transformation relationship between the robot twin's base coordinate system and end‐effector coordinate system, B_1_ can be converted to the end position. During navigation, the surgeon, wearing an AR device, visualizes the patient's tumor bed location and anticipates the direction of the skin incision. Through hand gesture interaction with B_1_, the end‐effector's posture is controlled to align with the tumor bed's spatial orientation, effectively preventing dangerous collisions between the robot and surgeons or patient.

As surgeon drags B_1_ in real‐time, the interaction adapts critical dose layer of the applicator with the spatial limiting plane of the personalized tumor bed, providing feedback through color changes to position the applicator at the optimal location. During this process, the function JointAngleDifferences is called to obtain the differences in joint angles *θ*_d_ of the twin units’ six joints before and after movement, acting as the incentive factor. This data is sent through the transit units to the physical entity units, updating the final joint angles’ values *θ*_f_ in the motion control panel of the physical robot using Equation (3).

(3) $$\theta_{f}=\theta_{s}+\theta_{d}=\left[\begin{array}{c} \theta_{f1}\\ \theta_{f2}\\ \theta_{f3}\\ \theta_{f4}\\ \theta_{f5}\\ \theta_{f6} \end{array}\right]$$

Once the angle updates are complete, the surgeon can perform forward movement of the physical robot, allowing the physical entity's applicator at the end‐effector to safely reach the target position. This completes the bidirectional driven control of the radiotherapy robot throughout the navigation process.

## 3 EXPERIMENTS DESIGN

### 3.1 Overview of user tests

We performed a series of experiments to test the effects of the hybrid twin kV‐IORT navigation system. Two navigation systems were developed for comparison. The first system we called the traditional kV‐IORT navigation system, where the mouse and keyboard were used as input tools, and the second system was the hybrid twin kV‐IORT navigation system. The differences between the two systems are shown in Table 2.

**TABLE 2:** The difference between the two navigation systems.

Category	Traditional IORT navigation system	IORT navigation system based on hybrid twin
Using method	Not immersive	Fully immersive with haptic feedback
Input module	Mouse and keyboard	Hand‐tracking interaction method
Interaction style	Keyboard to control the position of users in the virtual scene. Mouse to control the angle of view in the virtual scene.	Fully immersive interaction style. Users can walk around to observe any direction in the virtual scene. Hand tracking technology was used to interact with the virtual environment.
Haptic supported	Not supported	Supported
Navigating methods	Rough guidance	Personalized tumor bed area adaptive applicator positioning
Develop environment	Unity	Unity
Rendering device	2D computer screen	HoloLens rendering device

Abbreviation: IORT, intraoperative radiotherapy.

Both navigation systems were developed using Unity. These systems were based on the same surgical environment, including the overall operating room setup, medical equipment, and the preoperative personalized patient data. The main difference between them lies in the interaction methods and the corresponding navigation approach. The traditional kV‐IORT navigation system used a mouse and keyboard for interaction. Users moved within the virtual environment using the keyboard, while the mouse was used to control the viewpoint. The traditional system displayed the environment on a computer screen using common 2D rendering techniques, without tactile feedback. The user manually dragged the physical applicator to the tumor bed by aligning the virtual applicator and the virtual tumor bed on the screen, which is a common method in current kV‐IORT procedures.

Three steps were required for participants to perform in the experiment.

Step 1: Participants selected the appropriate spherical applicator type based on preoperative planning guidance.

Step 2: Participants used different navigation methods to position the robotic end‐effector's spherical applicator in the correct location relative to the personalized tumor bed.

Step 3: Participants simulated the kV‐IORT procedure on a phantom and validated the simulation results.

### 3.2 Participant sampling

Forty participants were selected for this study, divided into four groups based on occupation, age, and proficiency in kV‐IORT procedures. Each group consisted of 10 participants with varying levels of experience:

Medical school students (20–30 years): Familiar with kV‐IORT, possessing medical knowledge, and interested in new technologies like hybrid twin, but lacking practical surgical skills.

Medical school professors (35–50 years): Experienced in kV‐IORT procedures and skilled in surgery, with familiarity and quick adaptability to new technologies such as hybrid twin.

Unskilled doctors (20–30 years): Familiar with kV‐IORT but inexperienced in surgery and unfamiliar with new technologies, preferring traditional navigation methods.

Skilled doctors (35–50 years): Experts in kV‐IORT, with strong surgical skills but less familiarity with hybrid twin technology and slower adoption of new methods.

### 3.3 Procedure for the user test

The procedures of the user test involved the following three steps:

1. Preparation work before kV‐IORT. Two navigation systems were prepared for comparison: the traditional nonimmersive kV‐IORT system (using a personal computer) and the immersive hybrid twin system (using an AR device). A phantom was used for simulating radiotherapy and postoperative verification, with a treatment plan provided by experienced doctors.
2. Simulated kV‐IORT process. Participants were introduced to both navigation systems, including their differences and interaction methods. Each participant performed the kV‐IORT process using both systems, including selecting an applicator, registration, applicator navigation, and positioning.
3. Postoperative verification process. After positioning the applicator, participants performed a simulated skin incision and suturing on a phantom. Postoperative CT scans were taken to verify the applicator's positioning relative to the tumor bed inside the phantom. Participants then completed a satisfaction questionnaire and an interview on potential system improvements. The questionnaire included nine items, with the first seven using a seven‐point Likert scale (1 = strongly disagree, 7 = strongly agree). These seven questions assessed learnability (system impact on navigation skills) and efficiency (effectiveness of the hybrid twin's bidirectional driving mechanism). The final two questions addressed experimental errors based on the simulated procedure.

These questions were divided into two parts, namely, the feature of the system and the system efficiency. Moreover, two questions at the end of the questionnaire allowed participants to give their thoughts about this system.

Additionally, the System Usability Scale (SUS) questionnaire was used to validate the usability of the immersive navigation system.^25^ The participants were asked to complete this questionnaire as shown in Table 3 with “strongly disagree” for 1 and “strongly agree” for 5.

**TABLE 3:** Results of student's *t*‐test and one‐way ANOVA.

Group	Medical student		Medical professor		Unskilled doctor		Skilled doctor	
	Learna bility	Effect	Learnabi lity	Effect	Learnabi lity	Effect	Learnabi lity	Effect
*t*‐test p	0.00021	2.3E‐05	0.2742	2.7E‐05	6.1E‐09	2.5E‐05	0.156	0.00067

Category		Learnability	Efficiency
One‐wayANOVA	*F*	8.80121	31.249
	*p*	0.00399	0.00026

Abbreviation: ANOVA, A one‐way analysis of variance.

Student's *t*‐test was used to analyze the experimental data and examine the significant differences between the immersive and nonimmersive navigation.^26^ We can use Student's *t*‐test to verify the efficiency of the immersive system and evaluate its performance. Thus, we set the statistical significance level at *p* < 0.05. A one‐way analysis of variance (ANOVA) was performed using SPSS (IBM Inc.) to analyze the significant differences in learnability and system effect between the immersive and nonimmersive navigation systems among the four groups.

### 3.4 Experimental design of body model

**FIGURE 10:**
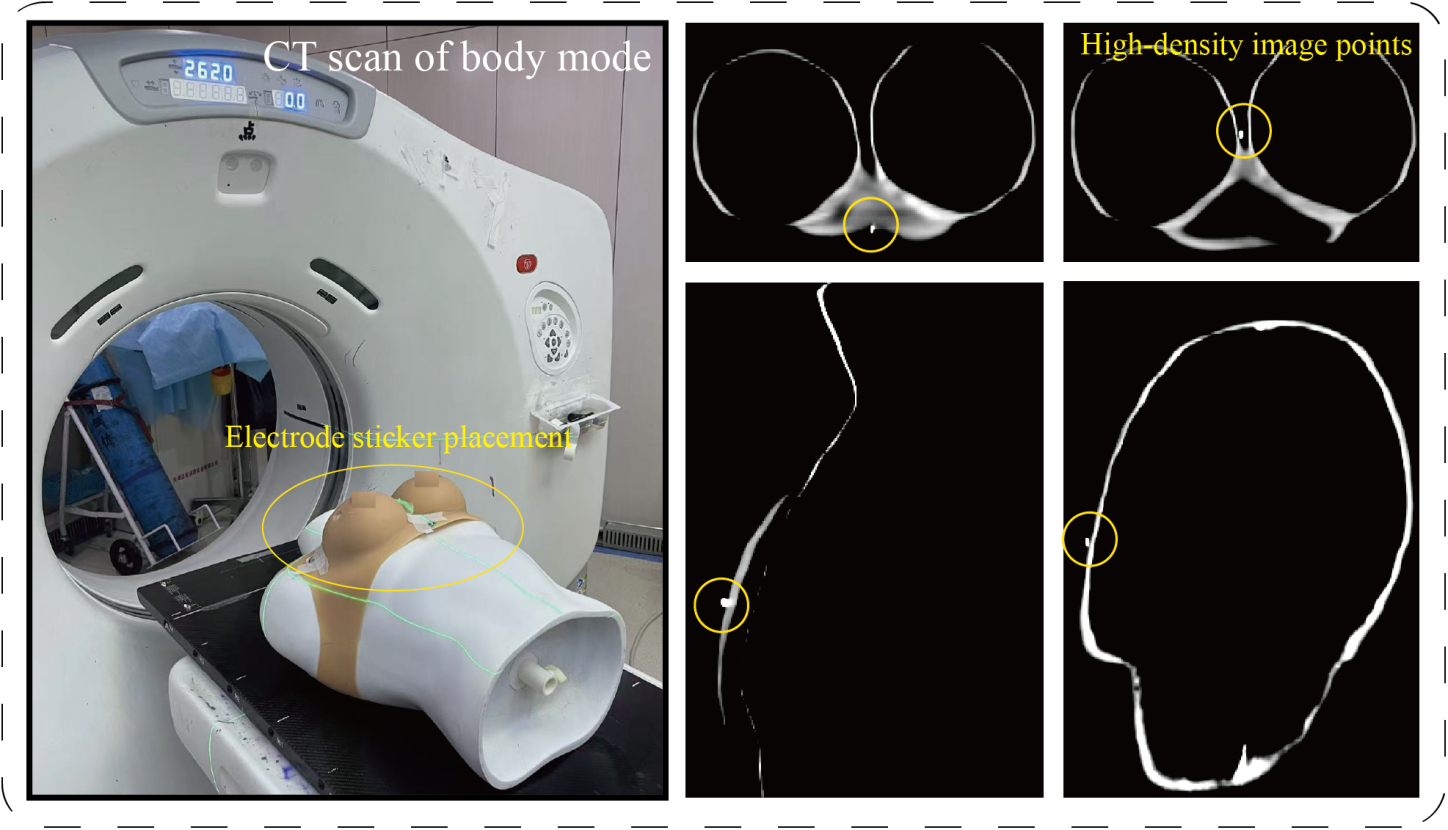
Representative illustration of fiducial markers in phantom scanning and CT imaging. CT, computed tomography.

The phantom experiment was conducted through CT‐guided irradiation under physician guidance. The tumor bed boundary curvature diameter was measured as 30 mm, with a 20 mm spherical applicator selected and optimal applicator positioning at 5 mm. Irradiation was administered for 25 min within the radiation dose range of 18.50–19.50 Gy. As shown in Figure 10, fiducial markers were systematically selected for CT scanning to facilitate phantom registration preparation.

We conducted multi‐module registration accuracy experiments to test the root mean square error (RMSE) between source point set V and target point set U during patient phantom and radiotherapy robot registration in hybrid space, while evaluating the reliability of error ranges through 95% confidence intervals to validate registration accuracy. In this experiment, we performed 20 test trials of phantom and radiotherapy robot registration in mixed reality, directly recording coordinates from their respective source point set V and target point set U.

**FIGURE 11:**
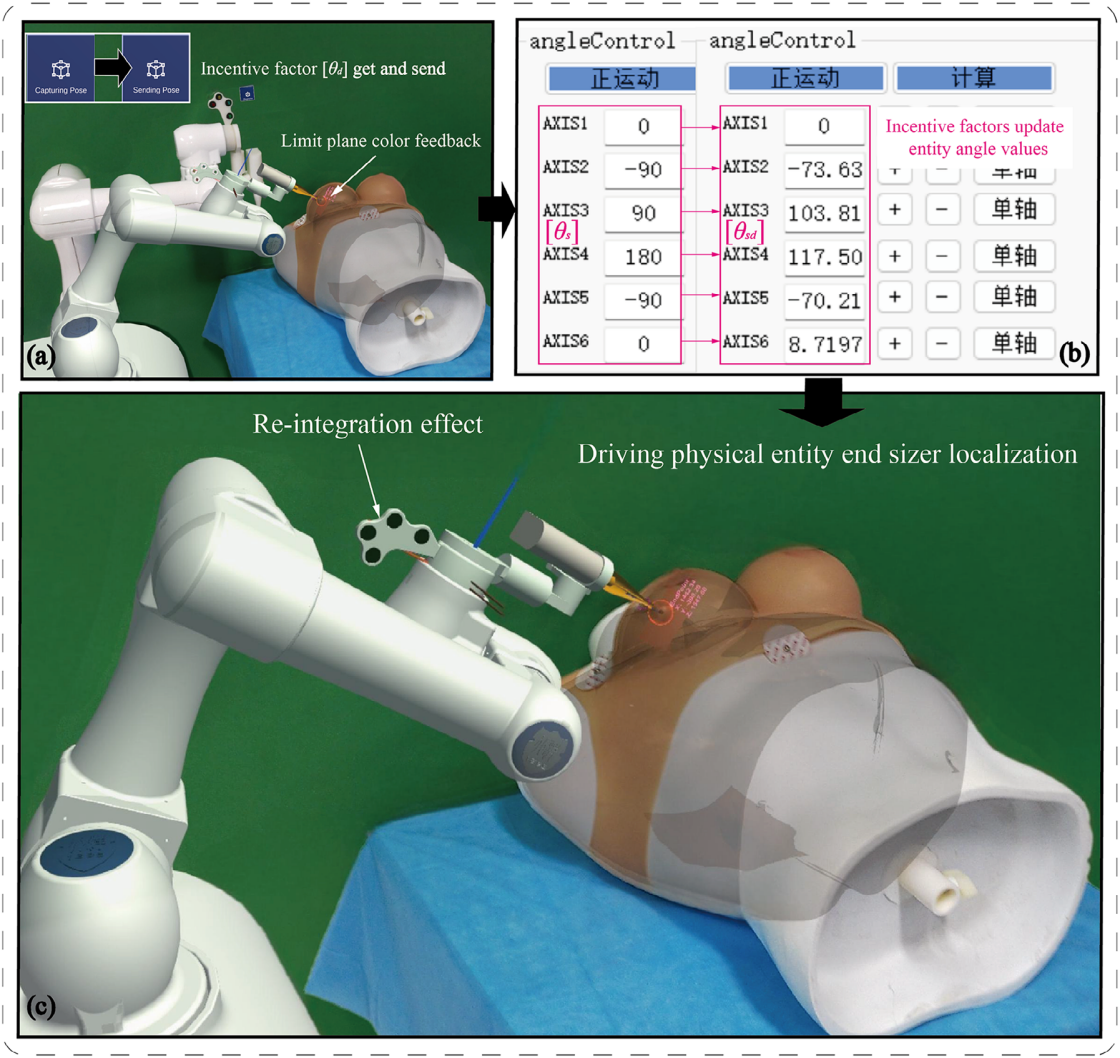
Experimental demonstration of robot physical entity driving and end‐effector positioning.

We conducted terminal applicator positioning accuracy experiments to verify whether the robot twin could precisely drive the physical entity's terminal applicator to reach the same target position within the tumor bed. The target registration error (TRE) was calculated using the spherical centroid coordinates of the applicator. Specifically, the spherical centroid coordinates $P_{e}$ of the physical robot's terminal applicator were directly obtained after positioning. A transformation matrix was derived using the centroid coordinates of four‐point fiducial markers in both physical and virtual spaces, enabling the calculation of $P_{v}$ (the corresponding standardized spherical centroid coordinates of the physical robot) based on the twin's current coordinates. The root mean square (RMS) value of TRE was computed across 20 experimental trials.As shown in Figure 11, the physician manipulated the registered virtual twin, and the virtual robot twin drove the physical robot's joint movements, enabling the applicator to reach the designated target area in the chest region of the phantom.

## 4 RESULTS

### 4.1 Results of the self‐constructed questionnaire

Table 3 indicates the differences in each group in learnability and efficiency‐based on *t*‐tests and one‐way ANOVA. The significance level was set at *p* < 0.05. The *P*‐value of the *t*‐test shows significance in the learnability items in the medical student and unskilled doctor groups but no significance in the medical professor and skilled doctor groups. The *P*‐value of the one‐way ANOVA of both learnability and effect among the four groups shows significance.

### 4.2 Results of the SUS questionnaire

**FIGURE 12:**
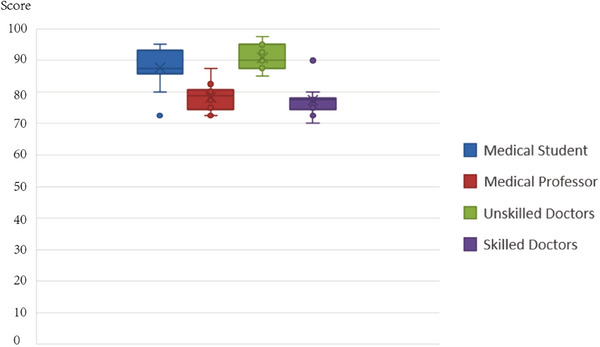
The results of the SUS questionnaire. SUS, System Usability Scale.

As shown in Figure 12 and Table 4, the different groups of participants had different responses to the navigation system. The average SUS score of the medical students was 87.75, and the Rank A percent of SUS was 90%. In the medical professor group, the average SUS score was 78.25, and the Rank A percent was 50%. Among the unskilled doctors, the average SUS score was 90.75, and the Rank A percent was 100%. Among the Skilled doctors, the average SUS score was 77.25, and the Rank A percent was 20%. The trend of the SUS results shows that the participants in Groups 1 and 3 gained better usability of this system, and Groups 2 and 4 showed limited usability of this system. In Groups 2 and 4, the participants were aged and worked for a long time with traditional kV‐IORT navigation, indicating that it was difficult for them to accept another new method of surgical navigation. Additionally, the comfort of the AR device may have increased their inconvenience. Among all participants, the average SUS score was 83.5, and the Rank A percent was 65%.

**TABLE 4:** Questionnaire for SUS.

Category	Content				
Effectiveness	I think that I would like to use this system frequently				
	I found the system unnecessarily complex				
	I thought the system was easy to use				
Efficiency	I think that I would need the support of a technical person to be able to use this system				
	I found the various functions in this system were well integrated				
	I thought there was too much inconsistency in this system				
	I would imagine that most people would learn to use this system very quickly				
Degree of satisfaction	I found the system very cumbersome to use				
	I felt very confident using the system				
	I needed to learn a lot of things before I could get going with this system				

Group	Max score	Min score	Average	Std	(Rank A) percent
Medical student	95	72.6	87.75	7.016	90%
Medical professor	87.5	72.6	78.25	4.721	50%
Unskilled doctor	97.5	85	90.75	4.090	100%
Skilled doctor	90	70	77.25	5.329	20%
Total	97.5	70	83.5	7.881	65%

Abbreviation: SUS, System Usability Scale.

### 4.3 Simulated surgery results

As shown in Table 5, the average accuracy in the simulated kV‐IORT of the medical students was 2.971 mm after kV‐IORT with the immersive system. The average accuracy of the unskilled doctors was 2.803 mm. Compared with the nonimmersive navigation system, the *t*‐test showed a *p*‐value lower than 0.05. The *t*‐test of the medical professors and skilled doctors shows a *p*‐value larger than 0.05, indicating that the immersive navigation system shows no significant difference between these two groups.

**TABLE 5:** Results of the error in simulated kV‐IORT navigation.

Group	Max error (mm)	Min error (mm)	Average (mm)	*t*‐test *P*‐value
Medical student	3.535	2.032	2.971	0.00015
Medical professor	1.890	1.302	1.450	0.274
Unskilled doctor	3.403	2.879	2.803	0.00038
Skilled doctor	1.580	1.309	1.438	0.483

Abbreviation: kV‐IORT, x‐ray intraoperative radiotherapy.

### 4.4 Results of body modeling experiments

**FIGURE 13:**
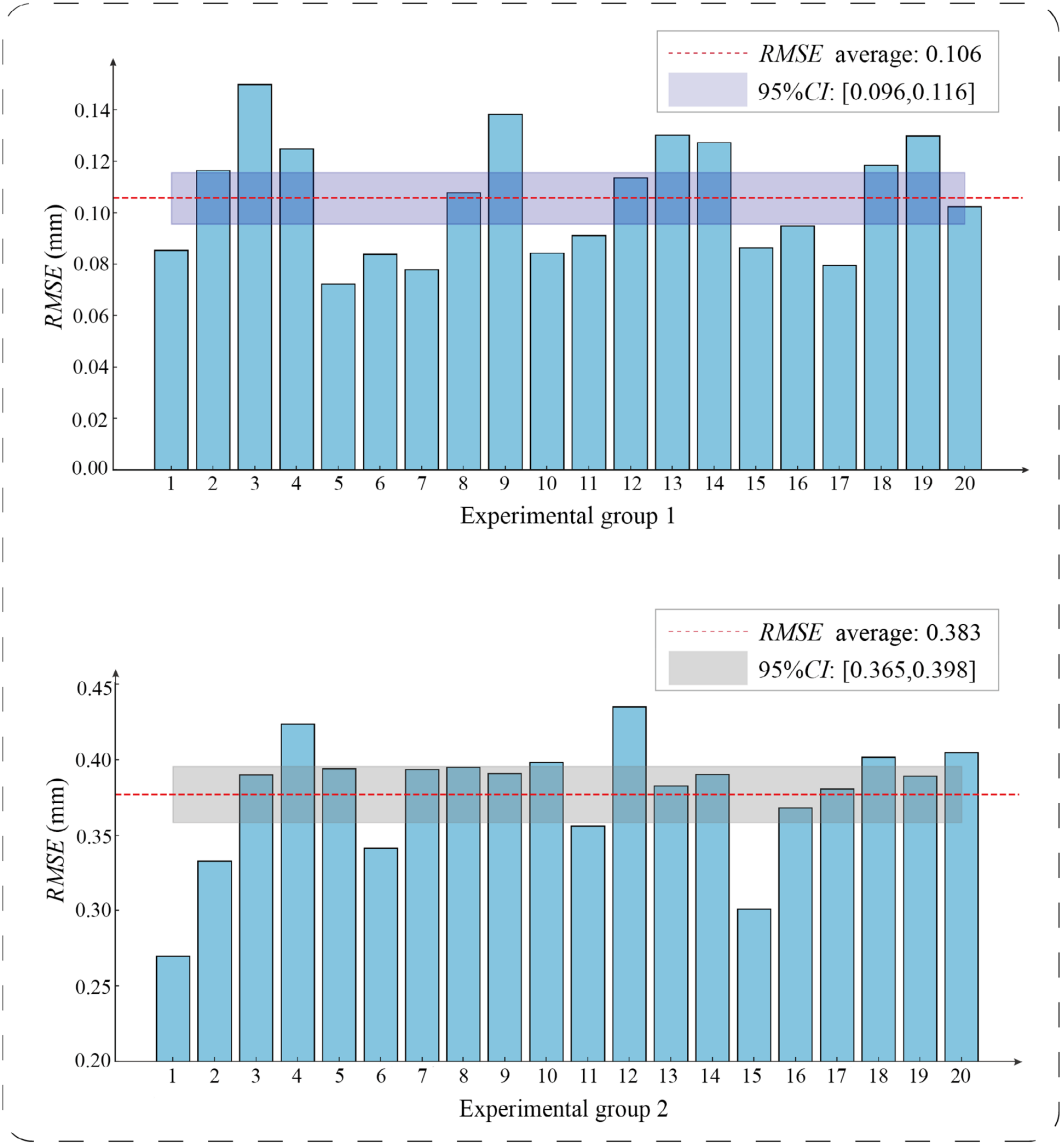
RMSE distribution of multi‐module registration accuracy experiments. RMSE, root mean square error.

As shown in Group 1 of Figure 13, the RMSE statistical samples for radiotherapy robot registration demonstrated a mean value of 0.106 with a standard deviation of 0.0208. Group 2 (phantom registration) recorded a mean RMSE of 0.383 and standard deviation of 0.0328. The registration error of the radiotherapy robot was 0.12 mm, while the patient phantom registration error reached 0.39 mm. These results meet the registration accuracy requirements for intraoperative radiotherapy navigation.

The end‐effector applicator positioning accuracy experiment ultimately yielded $TRE_{\mathrm{avg}}=0.41$ mm, which meets the intraoperative robotic positioning accuracy requirements. The experimental data are tabulated in Table 6.

**TABLE 6:** Results of the end‐effector applicator positioning accuracy experiment.

Groups	Navigational target	TRE/mm	Groups	Navigational target	TRE/mm
1	target area	0.42	11	target area	0.57
2	target area	0.26	12	target area	0.43
3	target area	0.51	13	target area	0.34
4	target area	0.37	14	target area	0.48
5	target area	0.28	15	target area	0.29
6	target area	0.45	16	target area	0.63
7	target area	0.32	17	target area	0.41
8	target area	0.39	18	target area	0.27
9	target area	0.54	19	target area	0.33
10	target area	0.35	20	target area	0.64

Abbreviation: TRE, target registration error.

## 5 DISCUSSIONS

Low‐energy kV‐IORT is a widely adopted technique for eliminating residual tumor cells and improving local control. However, precise positioning of the radiotherapy robot's applicator at the tumor bed is critical for optimal therapeutic outcomes. For less experienced surgeons, this task can be challenging, often relying on manual estimation, which increases the risk of suboptimal radiation delivery and compromised treatment efficacy. To address this, we proposed a multidimensional integrated hybrid twin navigation system that combines mixed reality and robotic twin technologies. The system is specifically designed to assist surgeons—particularly those with limited experience—in accurately and efficiently positioning the spherical applicator within personalized tumor beds.

As an immersive navigation tool, the hybrid twin system enables real‐time integration of digital and physical entities, facilitating faster learning and improved procedural fluency. To evaluate user perception and satisfaction, we conducted questionnaire‐based assessments. Many prior studies have explored navigation systems in brachytherapy and other surgical applications. For example, Sherry Zhao developed a cervical brachytherapy navigation system validated by the Asiatic Questionnaire^27^ which reported an average score of 4.5, indicating good usability for training. Vuthea Chheang introduced a virtual reality‐based liver surgery navigation system with an average SUS score of 77.5.^28^ The average SUS of our system was 83.5, highlighting its potential for usability improvements. Compared with Rothfusset al.'s six‐degree‐of‐freedom robotic arm IORT system (3.5 ± 0.6 mm positioning error),^29^ our hybrid twin system reduced operational endpoint errors to 2.97 mm through a multi‐source sensing network based on hybrid twin architecture demonstrating substantial accuracy improvements. Additionally, unlike conventional electromagnetic navigation systems requiring additional fiducial marker implantation,^30^ our approach achieves sub‐millimeter spatial matching through surface feature registration eliminating invasive marker placement. Innovatively, our system incorporates surgeons' spatial cognition into navigation constraints—a unique feature absent in purely technology‐driven systems—thereby bridging the gap between human expertise and machine automation. Additionally, the system's personalized navigation capabilities have increased its utility providing users with greater satisfaction and a sense of enhanced usability.

Analysis of self‐constructed questionnaire results showed a statistically significant difference (*p* < 0.05) in navigation accuracy between medical students and unskilled doctors using immersive versus non‐immersive systems. Younger participants, typically less experienced with traditional kV‐IORT workflows, were more receptive to immersive interfaces, which enhanced their learning and performance. In contrast, skilled doctors—already proficient in the procedure—showed minimal benefit from the immersive environment, as evidenced by similar performance metrics across both systems. This trend indicates that immersive systems may provide the greatest advantage to novices or those unfamiliar with kV‐IORT. As shown in Table 3, one‐way ANOVA further confirmed significant differences in both learnability and efficiency across participant groups (*p* < 0.05).

Additional qualitative feedback from participants (Table 7) reinforced these findings. When asked about their experience and suggestions, 26 participants reported that the immersive system improved familiarity and confidence in navigation. Seventeen cited discomfort from AR headset weight as a drawback. Most users appreciated the gesture‐based interaction, noting it as intuitive and more reflective of actual surgical movements. When asked what contributed to improved skill, participants frequently cited the immersive environment's focus‐enhancing qualities and its realistic simulation of navigation tasks. Only four users reported no improvement, and three reported dizziness from immersive use. Similarly, three users of the traditional system noted difficulty in control and adaptation. Notably, older users struggled more with adapting to gesture‐based interfaces, which may account for varied responses across age groups.

**TABLE 7:** Questionnaire for participants.

Category	Content
Learnability	Do you think this navigating method improves your IORT skills?
	Are you more confident after completing the navigating?
	Does the navigation system facilitate better concentration during surgery?
Efficiency	Are you satisfied with this system?
	Do you agree that you will have a clearer understanding of the process of IORT navigation after using this navigation system?
	Do you feel the interaction system is comfortable to use?
	Do you feel the navigating and positioning process is fast and comfortable?
Additional question	Please express your feelings about the two systems and the suggestions for improvement.
	If your navigating efficiency improved, please indicate the factors that led to a better result. If not, please explain why this system did not improve navigating efficiency.

Abbreviation: IORT, intraoperative radiotherapy

Post‐operative simulation results (Table 5) further supported the system's effectiveness. Statistical analysis using Student's *t*‐test showed significant improvements in surgical accuracy for Groups 1 (medical students) and 3 (unskilled doctors), but not for Groups 2 and 4 (professors and skilled doctors). Interestingly, despite the lack of significant differences in the latter groups, their baseline error values were lower, confirming their competence regardless of system type. These results suggest that immersive navigation systems can notably benefit novice users by enhancing surgical learnability and procedural efficiency.

Despite these promising outcomes, some limitations remain. The system's interface complexity may hinder operational fluency, as users must frequently switch between twin and physical views. In certain scenarios, the twin overlay can obstruct the surgical field, adding cognitive load. Furthermore, some users reported dizziness during navigation, often attributed to delayed system response and low frame rates. Future work will focus on simplifying the navigation interface for improved usability and optimizing twin model sizes to reduce memory load and maintain real‐time performance. Additionally, we aim to obtain clinical trial approval for radioactive comparative validations to comprehensively assess system accuracy and refine methodology.

## 6 CONCLUSION

This research presents a multidimensional integrated hybrid twin navigation system for kV‐IORT. The innovation of this research lies in the introduction of a Holo‐dose model specifically designed for personalized tumors in the body. This model visualizes the invisible radiation used in kV‐IORT, helping surgeons quickly determine the coverage area of key dose layers on the personalized tumor bed. Additionally, a hybrid twin bidirectional driving mechanism is established to achieve precise navigation of the radiotherapy robot's spherical applicator, driven by incentive factors.

## AUTHOR CONTRIBUTIONS

Shan Jiang: Conceived and designed the study, developed the multidimensional hybrid twin navigation system including its core modules and drafted the manuscript. Shuo Yang: Performed the phantom experiments, analyzed the data, and provided critical revisions to the intellectual content. Zhiyong Yang: Provided theoretical guidance and supervision. Daguang Zhang: Provided the key data for intraoperative radiology. Yingkai Luan: Assisted in the implementation of the augmented reality feedback module and validation of the applicator positioning strategy. Zeyang Zhou: Secured funding, supervised the overall project as the corresponding author, and reviewed the manuscript.

## CONFLICT OF INTEREST STATEMENT

The authors declare no conflicts of interest.

## ETHICS STATEMENT

This article does not contain any studies with human participants or animals performed by any of the authors.

## Supporting information



Supporting information

## APPENDIX I

### I.1

Maximum radial distance and minimum delivered dose values selected under different reference irradiation times. Divided into two categories: 20 mm applicator and 40 mm applicator. As follows:

**Table:**

20 mm Spherical Applicator			40 mm Spherical Applicator		
Reference exposure time/min	Maximum radial distance/mm	Minimum delivered dose/Gy	Reference exposure time/min	Maximum radial distance/mm	Minimum delivered dose/Gy
21	4	17.96	26	0.75	18.38
22	4.25	18.11	27	1	18.37
23	4.5	18.22	28	1.25	18.34
24	4.75	18.30	29	1.5	18.31
25	5	18.36	30	1.75	18.25
26	5.25	18.41	31	2	18.19
27	5.5	18.43	32	2.25	18.12
28	5.75	18.44	33	2.5	18.04
